# Learning about the changing needs for prosthetics service provision from routinely collected digital centre management data: An exemplar study across three clinics in Cambodia

**DOI:** 10.7189/jogh.12.04083

**Published:** 2022-10-30

**Authors:** Alex Dickinson, Lucy Gates, Cheryl Metcalf, Charlotte Owen, Sisary Kheng, Carson Harte, Sam Bunthoeun, Sam Simpson, Peter Worsley, Chantel Ostler, Maggie Donovan-Hall, Amos Channon

**Affiliations:** 1 Faculty of Engineering & Physical Sciences, University of Southampton, UK; 2 Institute for Life Sciences, University of Southampton, UK; 3 Exceed Research Network, Lisburn, UK; 4 Faculty of Environmental and Life Sciences, University of Southampton, UK; 5 Faculty of Medicine, University of Southampton, UK; 6 Centre for Global Health and Policy (GHAP), University of Southampton, UK; 7 Exceed Worldwide, Phnom Penh, Cambodia; 8 Exceed Worldwide, Lisburn, UK; 9 Portsmouth Hospitals University NHS Trust, Portsmouth, UK

## Abstract

**Background:**

Prosthetic service development and delivery rely on data describing population needs. These needs are context-specific, but most existing data come from high-income countries or small geographic areas, which are often not comparable. This study analysed routinely collected digital patient record data at multiple time points to provide insights into characteristics of people accessing Cambodian prosthetic services.

**Methods:**

We investigated trends in birth year, sex, year and reason for limb absence, and prosthesis type, over three decades. Then, we observed data from 2005 and 2019 indicating how the population actively accessing prosthetics services has changed.

**Results:**

Temporal trends in prosthetics service user demographics corresponded with events in Cambodia’s socio-political history. The predominant historical reason for limb absence prior to 2000 was weapon trauma during and following conflict. Since 2000, this was replaced by non-communicable disease and road accidents. Transtibial remained the most prevalent amputation level but transfemoral amputation had higher incidence for people with limb loss from road accidents, and people with limb loss due to disease were older. These observations are important as both transfemoral and older-aged groups experience particular rehabilitation challenges compared to the young, transtibial group.

**Conclusions:**

The study shows how standardised, routinely collected data across multiple clinics within a country can be used to characterise prosthetics service user populations and shows significant changes over time. This indicates the need to track client characteristics and provides evidence for adapting services according to population dynamics and changes in patient need.

Fifteen percent of the world’s population has a disability, over a billion people, and 80% live in lower and middle income countries (LMICs) [1]. The Global Burden of Disease study 2019 indicates the international prevalence of amputation at 176 million, which has increased by over 50% since 1990 [2]. The prevalence of major upper- and lower-limb amputations is estimated to be between 50 and 65 million [2], [3]. Established barriers to accessing rehabilitation services for people with limb absence include a lack of policy, standards and governance, affordability, service delivery systems, availability and production of prosthetic devices, and trained professionals [4].

In many LMICs, the high prevalence of amputation has resulted from conflict. In Cambodia, from 1979 to 2021, 19 779 people were killed and 45 144 people were injured by landmines, cluster munitions and other explosive remnants of war (ERW) following the Vietnam war, and subsequent civil conflict. This included 9067 people with amputations [5]. Approximately 5% of the Cambodian population has a disability which affects their walking or mobility, and 84% of people with disabilities in Cambodia live in rural communities and thus face barriers to accessing physical rehabilitation services [6]. Challenges faced by rehabilitation service providers in Cambodia include withdrawal of international aid, poor retention of experienced clinicians, and a lack of continuity of care from acute health care facilities into rehabilitation.

While long-term economic growth and technological development may improve prosthetics service provision the specific needs may change, and ability to deliver services may be disrupted. This may arise from gradual growth in non-communicable disease (NCD), or situations of conflict and natural disaster, where a sudden increase in need can occur alongside an interrupted ability to provide care. Such problems are often felt most acutely in LMICs. The demography of people accessing prosthetics services is expected to change considerably as landmines and ERW are cleared and urban environments grow rapidly, with an associated high incidence of road traffic accident injuries, especially involving young people using motorcycles [7,8]. Type 2 diabetes is also growing in prevalence [9], which elevates the risk of foot ulcers, infection and amputation [10]. This will likely necessitate a change in the specific requirements of prosthetics services, concerning prosthetic devices themselves, the provision and care pathway, and associated social support. For governments and non-governmental organisations (NGOs) to adapt their service provision in response to these changes they must be understood and predicted where possible, and this relies upon high quality data [11].

Prosthetics service need, access and delivery data are varied. Much of the existing data come from small geographic areas. The majority of available evidence on need has been generated through registry and health insurance data, which are concentrated in high income country (HIC) settings (eg, USA [12], UK [13,14] and Sweden [15]). Despite the high prevalence of disabilities and known barriers to access for rehabilitation services within LMICs, limited data are available to understand prosthetic services in these settings. The literature reports cross-sectional studies for low resourced settings such as Sierra Leone [16], Northern Uganda [17] and Malawi [18]. These set out to study social determinants of health amongst people with major limb loss, and identified barriers to access due to transport and health care service costs, and living in rural locations [18], as well as education and stigma [17] and a need for trained service provision staff and government support [16]. Other studies have taken a temporal approach, including two 1980s reports on a 24-year survey in Hong Kong [19] and a 15-year survey in Burma [20]. These studies identified trends in reasons for amputation and the numbers of people accessing prosthetic rehabilitation services as well as demographic disparities, most notably a considerably lower prevalence of prosthetic rehabilitation service use in women than men.

Household surveys can provide population-representative data, including those who do and do not access care, but they can be expensive and time consuming. However, digital patient records, compiled repeatedly for normal service purposes, provide a valuable and under-utilised resource as real-world evidence. The International Committee of the Red Cross (ICRC, Geneva) established a standardised digital patient management system (PMS) which offers an opportunity to identify and compare context-specific needs for prosthetics services at repeated time points, with homogeneous client demographics, standardised clinical data and outcome information. This data set has been used to study the demographics of people accessing ICRC physical rehabilitation services across 14 countries [21], and the demographics specifically of people with amputations in 5 countries [22].

Prosthetics service delivery has systems level challenges; service providers must ensure and evidence quality and provide continuity between health and rehabilitation services. In the context of growing NCD, the aim is early detection, intervention and prevention [23]. However, this can be complex when rehabilitation and health services are governed separately, as is the case in Cambodia. Successful service design depends on understanding the population’s needs in context, so we cannot rely solely upon evidence from high-income countries in order to develop sustainable services and inform workforce planning for LMICs. Facilitating service improvements and equitable provision, aligned with the WHO’s Universal Health Coverage agenda (UHC), therefore needs sustainable access to robust, routinely collected, accessible and meaningful data, that considers both functional outcomes and societal impacts and is comparable across services and locations. However, to date, there has been limited utility of this data.

This study aimed to provide novel insight into the demographics of those accessing prosthetic services in Cambodia and indicate their context-specific prosthetics service needs. Further, the study aims to show how Cambodian prosthetics service needs have changed over time and identify future service requirements.

## METHODS

An observational analysis of routinely collected longitudinal data over multiple time points was undertaken, to investigate the demographics of people accessing prosthetics services between 1992 and 2019 at three clinics run by charity Exceed Worldwide, in Phnom Penh, Kampong Som and Kampong Chhnang, Cambodia. These prosthetics services were provided alongside orthotics services by dual-qualified staff. Approval was granted by national (230&311NECHR) and institutional ethics review boards (ERGO45577&51898) to analyse episodal statistics extracted from Exceed’s digital clinical records. Records were collected in a standardised manner and stored in the “PMS-5” database (ICRC, Geneva, Switzerland) for all individuals or “clients” accessing Exceed’s clinics for prosthetics services from 1998 until December 31, 2019. The earliest clinical contact recorded within this data set was the December 2, 1992 in Phnom Penh, February 14, 1993 in Kampong Som and September 14, 1995 in Kampong Chhnang. Records from before 1998 were input by the clinics from paper records for specific individuals whose care was ongoing. Each line of data described a single clinical contact such as an assessment, a prosthetic device provision, a replacement, or a repair. An individual could have multiple contacts for provision of prosthetic devices, and an individual device could have multiple repairs. Individuals were considered “active” if they had at least one appointment in the last 7 years, on the Exceed Country Director’s recommendation (SK).

Data analysis was performed in Stata (v16, StataCorp, Texas, USA), to describe the demographics of clients accessing the prosthetics service and the devices they were prescribed, including the client’s year of birth, gender, year of limb absence or amputation, reason/cause of limb absence/amputation, and type of prosthesis as a proxy for limb absence level. Descriptive statistics were extracted at 6 time points to assess the temporal trends in these demographics, and χ^2^ tests were performed to assess the null hypotheses i) that each demographic category was the same for “all” and “active” groups, and ii) that each demographic category did not change over time. Hypothesis tests of independent sample proportions were conducted to assess demographic differences between particular groups and the rest of the study population. Finally, two exemplar cross-sectional analyses were reconstructed from the data set, representing the active clients at the end of 2005 (ie, the earliest cross-section based on clients with fully digital records, 7 years after 1998) and the end of 2019 (ie, the latest cross-section at time of data extraction). χ^2^ tests were performed to assess the null hypotheses that demographic categories were the same for the reconstructed cross sections.

## RESULTS

### Client Demographics

After removing duplicate appointments, the data set contained 50 144 entries representing clinical contacts for 7117 individual clients. Of these clients, 2820 were classified as active. Most clients had at least partial data recorded. 351 individuals (4.9%) did not have any data apart from the date of the initial assessment appointment. Person characteristics for all and active clients, including reason for limb loss and type of prosthetic supplied, are shown in **Table 1**. The majority of prostheses supplied were for lower limb absences, with transtibial and transfemoral devices supplied in a ratio of 5:1 for both all and active clients. Leaving out “missing” data, the predominant reason for limb absence was amputation after weapon injuries (77%) (including landmines, enhanced radiation weapons, grenades or gunshots), followed by road traffic accidents (6.9%) and a range of illness or disease-related causes (5.6%). χ^2^ tests revealed differences in the distribution of all demographics between “all” and “active” client groups (χ^2^≥9.85, *P* < 0.01) except for side (χ^2^ = 0.263, *P* = 0.8768).

**Table 1:** Raw demographics of people accessing Exceed Worldwide services for prosthetic assessment, prosthetic device provision, repair, and replacement

		All Clients (%), n = 7117	Count	Active Clients (%), n = 2820	Count
Sex	Female	11.8	838	13.7	387
	Male	83.3	5928	78.9	2224
	Missing	4.9	351	7.4	209
Side	Left	45.2	3215	44.0	1242
	Right	45.7	3253	43.7	1233
	Bilateral	2.6	185	2.6	74
	Missing	6.5	464	9.6	271
Clinic	Phnom Penh	44.9	3198	34.8	981
	Kampong Chhnang	25.5	1814	36.1	1017
	Kampong Som	29.6	2105	29.2	822
Year of birth (age at end 2019, years)	Before 1940 (>79)	3.0	210	1.2	35
	1940-1959 (60-79)	25.5	1817	23.6	664
	1960-1969 (50-59)	42.0	2987	38.9	1098
	1970-1979 (40-49)	14.7	1049	12.5	351
	1980-1989 (30-39)	5.7	408	8.5	240
	1990 and later (≤29)	4.2	295	7.9	223
	Missing	4.9	351	7.4	209
Age at first consultation (years)	0-19	5.8	413	7.9	224
	20-29	19.3	1370	16.6	468
	30-39	34.5	2455	29.0	817
	40-49	20.5	1459	20.7	583
	50-59	9.7	689	12.6	354
	60+	5.3	380	5.9	165
	Missing	4.9	351	7.4	209
Reason for limb absence / cause of amputation	Congenital	2.9	203	3.9	110
	Road traffic accident	5.0	358	8.3	235
	Weapon injury	56.7	4033	55.1	1554
	Animal bite	0.7	46	1.2	33
	Illness*	4.1	293	5.1	145
	Accident at work	2.1	149	2.5	70
	Other	1.9	135	3.0	84
	Missing	26.7	1900	20.9	589
Type of prosthesis supplied, as a proxy for level of limb absence or amputation	Partial foot	2.5	179	2.3	65
	Transtibial (all)	61.6	4385	63.5	1790
	Knee disarticulation	1.0	68	1.2	34
	Transfemoral	22.0	1564	19.5	549
	Transradial	4.8	343	3.4	97
	Transhumeral	1.5	105	1.3	37
	Other	1.7	122	1.4	39
	Missing	4.9	351	7.4	209

*Illness for all clients includes diabetes (17.1%), disease (41.6%), gangrene (5.1%), infection (35.5%). Illness for active clients includes diabetes (28.3%), disease (47.6%), gangrene (1.4%), infection (21.4%).

**Figure 1:**
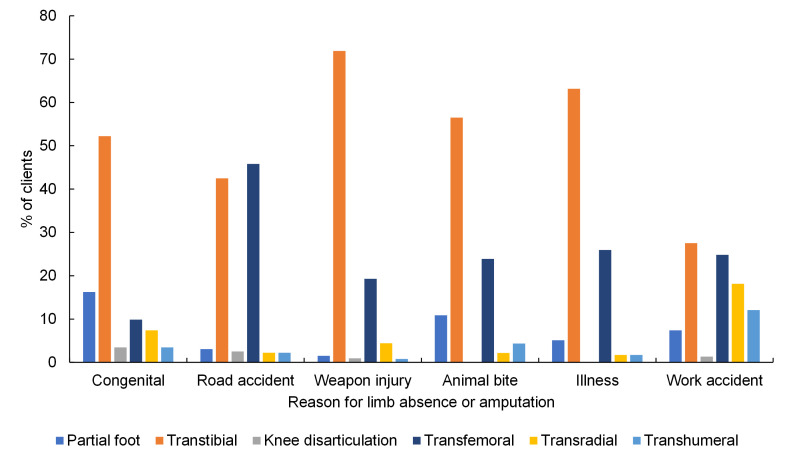
Percentage of types of prosthetic device supplied, as a proxy for level of limb absence or amputation, for each reason for limb absence or amputation, for all clients, omitting “other” and “missing” categories (n = 5082).

Weapon injury was the most common reason for limb absence at all levels (Table S1 in the **Online Supplementary Document**). Noteworthy proportions of transfemoral amputations were linked to road traffic accident, partial foot amputation or absence for congenital reasons, and upper limb absence for congenital and accidents at work (**Figure 1**). The most common absence level was transtibial for all reasons except road traffic accidents, for which transfemoral devices were most common (**Figure 1****,** Table S1 in the **Online Supplementary Document**). Transfemoral devices were more common for road traffic accidents than for other reasons of limb absence (*P* < 0.001). Upper limb devices were considerably more common for people with limb loss following accident at work (30%) vs other reasons for absence (*P* < 0.001). Partial foot absence represented 16% of people with congenital limb absence and 11% of people with amputation following animal bite, which was more common than for other reasons for limb absence (both *P* < 0.001).

**Figure 2:**
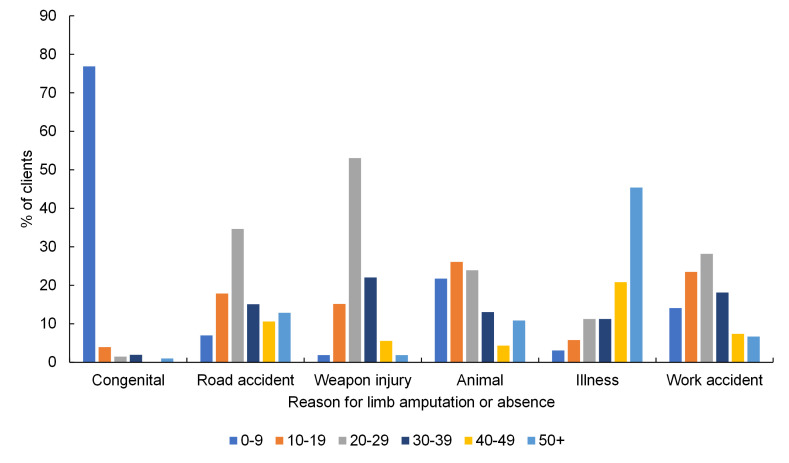
Percentage of clients grouped by age at amputation or limb absence, for each reason for limb absence, for all clients, omitting “other” and “missing” categories (n = 5082).

The most common band of age at limb absence or amputation due to road traffic accident, weapon injury and accident at work was 20-29 years (**Figure 2****,** Table S2 in the **Online Supplementary Document**). A notable difference was amputation due to illness, which showed a markedly increasing trend across age groups. The majority of people listed as having congenital limb absence or amputation experienced this when aged between 0 and 9 years. Amputation due to congenital limb absences or amputations, and due to animal bite were more commonly experienced by younger people (0-19 years) than the other amputation reasons (both *P* < 0.001).

### Temporal Trends

**Figure 3:**
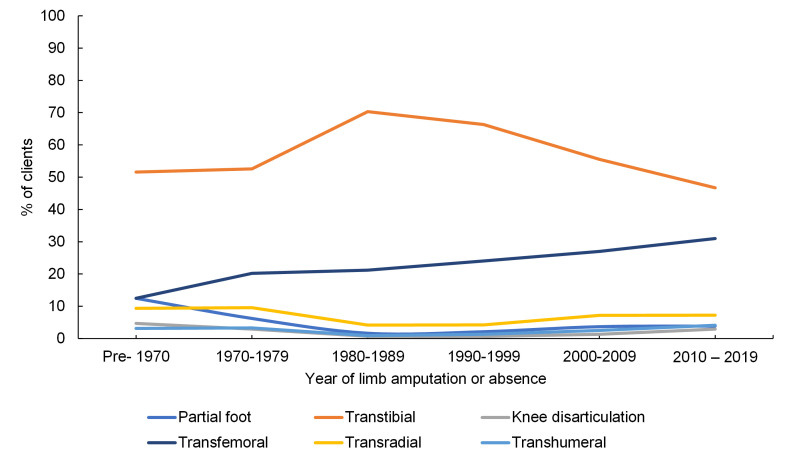
Percentage of level of limb absence for the clients’ year of amputation or absence grouped by decades, for all clients, omitting “other” and “missing” categories (n = 6644).

**Figure 3** and Table S3 in the **Online Supplementary Document** show the change of supply in seven different prosthetic devices, as a proxy for level of limb absence, by the year of absence or amputation. The main trends were a proportional decline in people with transtibial absences from a peak in the 1980-89 decade (χ^2^ = 11.3, *P* = 0.01), and a proportional rise in transfemoral absences over the full period (χ^2^ = 65.7, *P* < 0.001). All other levels of limb absence remained below 12.5%.

**Figure 4:**
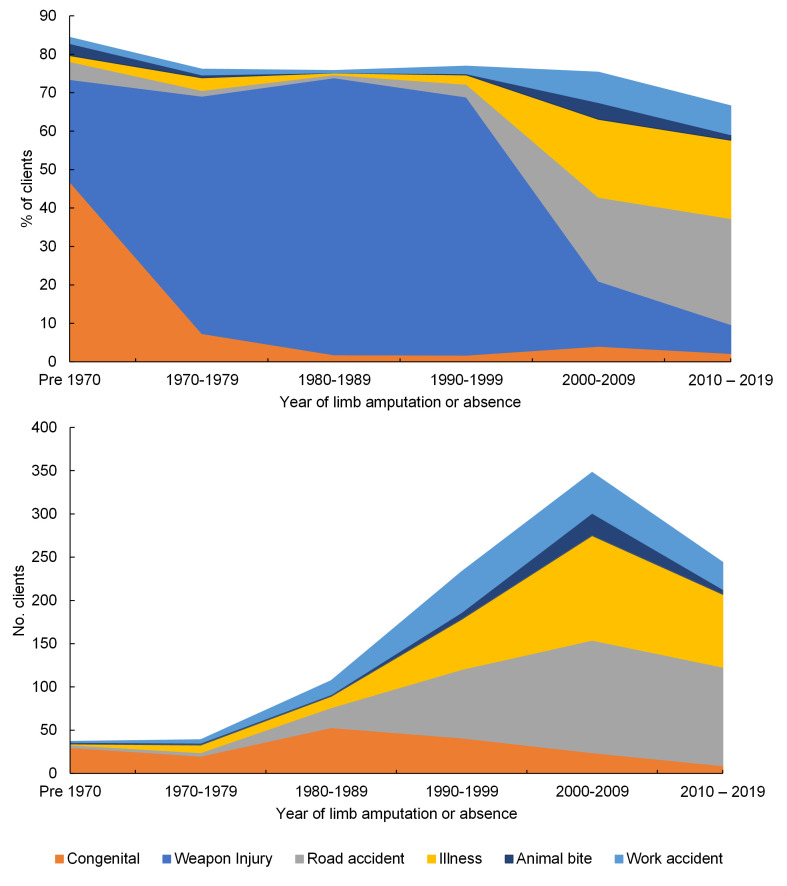
Top: Percentage of reason for limb amputation or absence for the clients’ year of amputation or absence grouped by decades, for all clients (n = 5082). The remaining % is an increasing proportion of “missing” or “other” categories. Bottom: the number of clients with each reason for limb amputation or absence per decade, omitting “weapon injury”.

**Figure 4** and Table S4 in the **Online Supplementary Document** show the temporal trends in frequency of reason for limb absence over six decades between <1970 and 2019 for all clients. Notably the proportion of people experiencing amputation following weapon injury peaked in the 1980-89 decade, and since 2000 road traffic accident and trauma have become the most common causes of limb absence (*P* < 0.001).

**Figure 5:**
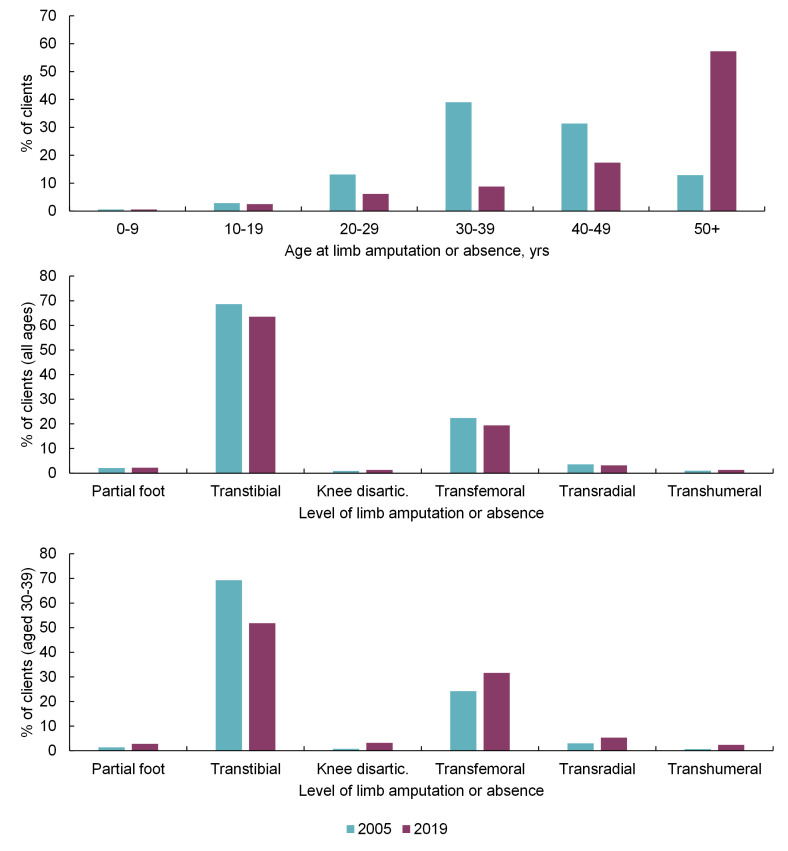
Top: Age distribution of clients actively accessing the service in 2005 and 2019. Of these clients, Middle: the distribution of levels of limb absence or amputation omitting “other” and “missing” categories, for all clients (2005, n = 5170; 2019, n = 2820), and Bottom: for those in the exemplar 30-39-year-old age group (2005, n = 2015; 2019, n = 247).

**Figure 5** and Table S5 in the **Online Supplementary Document** show how the age distribution and level of limb absence or amputation (%) differ with age for clients who were classified as active in 2005 and in 2019. The existing, active population accessing this service has become older (**Figure 5** top, χ^2^ = 2109, *P* < 0.001). While the overall distribution of absence levels has changed little between 2005 and 2019 (**Figure 5** middle, χ^2^ = 8.56, *P* = 0.128), inspecting subgroups shows significant differences with a proportional decrease in the transtibial level in the 10-49-year-old age groups (example in **Figure 5** bottom: χ^2^ = 40.9, *P* < 0.001). The distribution of absence levels in the 50+ year age group was unchanged in 2019 compared to 2005.

## DISCUSSION

Using routinely collected data this study provides insight into the clinical and demographic characteristics of people who have experienced limb absence or loss in Cambodia over more than six decades. By presenting temporal trends in patient characteristics and prosthetic device types, this study indicates how the Cambodian prosthetics service needs have changed over time, based on reasons for limb absence and the patients’ demographics. In Cambodia the prevalence of landmine and ERW injuries is understood anecdotally, but researchers report a mismatch between in-country data and published literature [24]. The design and delivery of prosthetic services would benefit from understanding key patient descriptors of age, level and reason for limb absence, allowing the adjustment of resource allocation, service planning and delivery, to maintain and improve care and to target investment in services where it is most needed.

The present study facilitated an overall description of the people accessing prosthetics services in Cambodia (**Table 1****,** Table S1-S2 in the **Online Supplementary Document****,** **Figure 1** and **Figure 2**). Temporal information was obtained by combining groupwise analyses with their changes over more than three decades, including the proportion of clients accessing the service for different prosthetic devices (**Figure 3****,** Table S3 in the **Online Supplementary Document**), and for different causes of limb absence (**Figure 4****,** Table S4 in the **Online Supplementary Document**). This revealed insightful temporal trends in the demographics of people undergoing amputation which correspond to known historical events, and the devices prescribed to them.

The data set shows an overall decline in incidence of limb amputation or absence since a peak in the 1980s, dominated by the trend in weapon injuries. However, there was an underlying rise in the absolute number of amputations linked to road traffic accidents, illness, and accidents at work throughout the period to a peak in 2000-09. For the small group of clients experiencing limb absence or amputation pre-1970 (n = 64), congenital defects predominated, believed to be linked to the defoliant Agent Orange used during the Vietnam War (1961-71) [25]. The data set shows a declining number of clients with limb absence or amputation for congenital reasons since the 1980s. A dominant factor was the rise in weapon injuries from pre-1970s until a gradual decline in 1990s (**Figure 5**, Table S5 in the **Online Supplementary Document**). This trend corresponds with the end of the Vietnam War (1961-71), the Cambodian Civil War (1968-75) and the Khmer Rouge regime (1975-89) and subsequent Cambodian-Vietnamese War (1979-89), with peace and political stability generally considered to have been reached in 1999. The predominance of weapon injury as the reason for amputation reduced markedly from 2000-2009 onwards and was replaced with a wider variety of causes including road traffic accident and illness (primarily listed as diabetes, infection, or gangrene).

The transtibial absence level clearly had the highest incidence in all decades (**Figure 3****,** Table S3 in the **Online Supplemetary Document**) but has declined since the 1980-89 decade, and there is a gradual increase in the proportion of transfemoral absences. Possible explanations may be found in the changing reasons for limb absence, most notably the increasing incidence of amputation following road traffic accidents. Traumatic injury in road traffic accidents has a historic burden and is a growing cause of lower extremity loss in many LMICs [26]. The growth in vehicle numbers, insufficient law enforcement, lack of road safety education, speed increases and inadequacy of health services have led to a rapid rise in road fatalities and injuries [27], and 7/10 injuries and deaths in 2014 were attributed to road accidents [6]. The increasing prevalence of transfemoral amputation may correspond with the different form of traumatic injury sustained in a road accident, especially for motorcyclists and passengers in side-on collisions with cars. Individuals with transfemoral absence are known to have more complex rehabilitative needs and poorer outcomes [28], and require more complex devices that take longer to manufacture and are more challenging to maintain.

Therefore, information generated in this study provides direction for future areas of prosthetic device research and development, and indicators for forthcoming service delivery needs. These temporal trends in the data were filtered for active clients to create cross-sections at the end of 2005 (ie, clients who were active in the 7 years since the digital record began) and at the end of 2019, to allow comparison of the demands upon service providers at particular time points (Table S5 in the **Online Supplementary Document**). The total number of clients has reduced, though their age profile has increased (**Figure 5** top). The ageing of existing clients, and entry of new older clients with amputations most commonly due to illness as observed by Barth et al. [22], appears to be outweighing the entry of new young clients into the service. This will impact the complexity of patient management and the ability of the government and NGOs to deliver the same interventions within existing timeframes. This may indicate a need for more holistic management as needs become more complex. Looking at the whole group there has been very little apparent change in the distribution of clients’ level of limb absence (**Figure 5** middle), although the decline in the proportion of transtibial and increase in transfemoral absence is noticeable in younger age groups, (**Figure 5** bottom). Interestingly there was little change in the limb absence level for people aged 50 and above between the 2005 and 2019 cross sections. Illness was a common cause of amputation in this age group (**Figure 2**), so a partial explanation may be the rising prevalence of NCDs and associated comorbidities such as diabetes, which is rising fastest in LMICs [9]. Diabetes is a known risk factor for lower limb amputations and presents an associated societal burden [10]. Mortality studies in high resourced settings [29] indicate median life expectancy of 20 months following major lower limb amputation for vascular or infection-related reasons, and 77% mortality at 5 years. In this data set only 17.1% of amputations due to illness were linked specifically to diabetes, and a further 35.5% to infection, 5.1% to gangrene and 41.6% to unspecified disease. The high proportion of unspecified disease may indicate different conventions of reporting and could indicate an under-estimate of the true level of diabetic causes. The impact of any associated comorbidities will further accentuate these figures, and this may give an insight into future challenges with rising rates of amputation due to NCD in low resourced settings.

This study is limited by reliance on secondary analysis of clinical data sets, which represent only partial national coverage for three of eleven clinics. Its accuracy was dependent on reporting practices and is influenced by any differences in data input regimes between the three centres, and some data were missing. These clinics serve clients across a wide range of the population in Cambodia both in urban and rural regions, free of charge, so while the study’s generality cannot be confirmed, the findings are not restricted by socioeconomic status and therefore broadly reflect the wider population. There may also be cohort effects associated for example with the changing survivorship of trauma, development and treatment of non-communicable disease, and the proportion of clients characterised as inactive. The study provides no information on people in need who do not access physical rehabilitation services, and no reason for why people stop appearing in the data set. This may be due to death, people moving and accessing a different prosthetics service, or ceasing to use their prosthesis. Some people may have accessed more than one centre, which could only be accounted for if the data set noted a national identification record. Future work might investigate the declining number of people accessing prosthetics services in the last decade, and whether this is due to the existing population reaching their life expectancy, standardised prosthetic devices lasting longer or no longer meeting the needs of an emerging middle class who then choose to access services elsewhere, or people experiencing more difficulty in accessing services due to reducing financial support. Further consideration of dimensions including sex should be made, noting that female clients are in a clear minority in this and similar data sets [19,20,22]. Findings might enhance our recommendations for improving the equity and diversity of the service. Finally, details were available on a variety of service delivery factors, but the present study’s scope was limited to the demographics of those accessing the service, and we do not consider functional outcomes or societal impacts.

## CONCLUSIONS

Routinely collected clinical data are regularly used in high income countries, but less in LMICs, partly because data are not so easily available, and partly due to the dearth of professionals to analyse them, and necessary focus on service delivery above all [30]. This first observational study of people accessing prosthetics clinics in Cambodia emphasises the benefits of using routinely-collected longitudinal clinical data across multiple prosthetic clinics and highlights the importance of having standardised data on aetiology, management and outcomes. It supports the International Society for Prosthetics & Orthotics (ISPO)’s work to identify the core requirements for a global registry, and ATscale whose 2020 Product Narrative report for Prostheses proposed that “defining the core dataset of amputee data and outcome measures will underpin the efforts of countries to implement registries” and “Creation of a global platform and governance for aggregation of country-level data will enable consolidated insights” [11]. This study presents an exemplar dynamic analysis which could be implemented by any centre or country using the ICRC’s PMS, the most widely used digital record in low-resourced settings, and potentially their new Digital Centre Management System (DCMS) as it is rolled out [31]. It provides a call-to-arms for comparable studies across other contexts, for example to extend the general physical rehabilitation service research on multi-country data [21]. These findings show the potential for registry data to identify the essential value delivered by prosthetics services and extract evidence to help service providers share best practice and adapt to a changing population and dynamic patient needs, and may be extended to other causes of disabling conditions.

## Additional material


Online Supplementary Document
