# Supplementary material for: Learning about the changing needs for prosthetics service provision from routinely collected digital centre management data: An exemplar study across three clinics in Cambodia
Source: J Glob Health. 2022 Oct 30;12:04083. doi: 10.7189/jogh.12.04083 (PMC9579830; doi:10.7189/jogh.12.04083)
Supplement: Online Supplementary Document [file jogh-12-04083-s001.pdf]

ONLINE SUPPLEMENTARY DOCUMENT

Title: Learning about the changing needs for prosthetics service provision from routinely collected digital centre management data: an exemplar study across three clinics in Cambodia

Authors: A Dickinson, L Gates, C Metcalf, C Owen, S Kheng, C Harte, B Sam, S Simpson, P Worsley, C Ostler, M Donovan-Hall & A Channon

Table S1: Data behind Figure 1: percentage of reason for limb absence for each type of prosthetic device supplied, as a proxy for level of limb absence, for all clients (n=3451). Percentages are calculated in column categories.

	**Partial Foot**	**Transtib**	**Knee Disartic**	**Transfem**	**Transrad**	**Transhum**	**Other**	**Missing**	**Count**
Congenital	18.4	2.4	10.3	1.3	4.4	6.7	12.3	0.0	203
Road Traffic Accident	6.2	3.5	13.2	10.5	2.3	7.6	4.9	0.0	358
Weapon Injury	34.1	66.1	55.9	49.7	51.9	30.5	38.5	0.0	4033
Animal Bite	2.8	0.6	0.0	0.7	0.3	1.9	0.8	0.0	46
Illness	8.4	4.2	0.0	4.9	1.5	4.8	5.7	0.0	293
Accident at Work	6.2	0.9	2.9	2.4	7.9	17.1	10.7	0.0	149
Other	0.6	1.3	4.4	1.8	7.6	7.6	10.7	0.0	135
Missing	23.5	21.0	13.2	28.8	24.2	23.8	16.4	100.0	1900
Count	*179*	*4,385*	*68*	*1,564*	*343*	*105*	*122*	*351*	*7,117*

	**Congenital**	**Road Traffic Accident**	**Weapon Injury**	**Animal Bite**	**Illness**	**Accident at Work**	**Other**	**Missing**	**Count**
Partial Foot	16.3	3.1	1.5	10.9	5.1	7.4	0.7	2.2	*179*
Transtibial	52.2	42.5	71.9	56.5	63.1	27.5	41.5	48.4	*4385*
Knee Disarticulation	3.5	2.5	0.9	0.0	0.0	1.3	2.2	0.5	*68*
Transfemoral	9.9	45.8	19.3	23.9	25.9	24.8	20.7	23.7	*1564*
Transradial	7.4	2.2	4.4	2.2	1.7	18.1	19.3	4.4	*343*
Transhumeral	3.5	2.2	0.8	4.4	1.7	12.1	5.9	1.3	*105*
Other	7.4	1.7	1.2	2.2	2.4	8.7	9.6	1.1	*122*
Missing	0.0	0.0	0.0	0.0	0.0	0.0	0.0	18.5	*351*
Count	*203*	*358*	*4,033*	*46*	*293*	*149*	*135*	*1,900*	*7,117*

Table S2: Data behind Figure 2: percentage of clients grouped by age at amputation or limb absence, for each reason for limb absence, for all clients (n=3541). Percentages are calculated in column categories.

Cause	Congenital	Road Traffic Accident	Weapon	Animal	Illness	Work Accident	Other	Missing	Count
0-9	76.9	7.0	1.9	21.7	3.1	14.1	6.7	2.3	349
10-19	3.9	17.9	15.2	26.1	5.8	23.5	17.8	11.3	986
20-29	1.5	34.6	53.0	23.9	11.3	28.2	26.7	38.7	3123
30-39	2.0	15.1	22.0	13.0	11.3	18.1	8.2	15.4	1317
40-49	0.0	10.6	5.6	4.4	20.8	7.4	12.6	5.2	451
50+	1.0	12.9	1.9	10.9	45.4	6.7	12.6	6.2	406
Missing	14.8	2.0	0.5	0.0	2.4	2.0	15.6	21.0	485
Count	*203*	*358*	*4,033*	*46*	*293*	*149*	*135*	*1,900*	*7,117*

Table S3: Data behind Figure 3: percentage of types of prosthetic device supplied, as a proxy for level of limb absence, for the clients' year of amputation or absence grouped by decades, for all clients. Percentages are calculated in column categories.

	**Pre-1970**	**1970-1979**	**1980-1989**	**1990-1999**	**2000-2009**	**2010 – 2019**	**Missing**	**Count**
Partial Foot	12.5	6.3	1.7	2.1	3.7	3.9	3.6	179
Transtibial	51.6	52.6	70.3	66.3	55.5	46.7	12.3	4385
Knee Disarticulation	4.7	2.9	0.7	0.7	1.3	2.9	0.2	68
Transfemoral	12.5	20.2	21.2	24.1	27.0	31.0	4.6	1564
Transradial	9.4	9.6	4.2	4.2	7.2	7.3	3.1	343
Transhumeral	3.1	3.3	0.9	1.3	2.5	4.1	0.6	105
Other	6.3	5.2	1.1	1.3	2.7	4.1	2.1	122
Missing	0.0	0.0	0.0	0.0	0.0	0.0	73.4	351
Count	*64*	*272*	*2,904*	*2,390*	*596*	*413*	*478*	*7,117*

Table S4: Data behind Figure 4:percentage of reason for limb amputation or absence for the clients' year of amputation or absence grouped by decades, for all clients. Percentages are calculated in column categories.

	Pre 1970	1970-1979	1980-1989	1990-1999	2000-2009	2010 – 2019	Missing	Count
**Congenital**	46.9	7.4	1.8	1.7	4.0	2.2	5.4	203
**Road Traffic Accident**	4.7	1.5	0.8	3.3	21.8	27.6	1.1	358
**Weapon Injury**	26.6	61.7	72.1	67.2	17.0	7.5	3.8	4033
**Animal Bite**	3.1	0.7	0.07	0.3	4.4	1.5	0.0	46
**Illness**	1.6	3.3	0.5	2.4	20.3	20.3	1.5	293
**Accident at Work**	1.6	1.5	0.6	2.0	7.9	7.5	0.6	149
**Other**	3.1	1.1	0.2	1.0	6.4	9.9	4.4	135
**Missing**	12.5	22.8	24	22.1	18.3	23.5	83.3	1900
Count	*64*	*272*	*2,904*	*2,390*	*596*	*413*	*478*	*7,117*

Table S5: Data behind Figure 5: percentage of the type of prosthetic device supplied, as a proxy for level of limb absence, for the clients' current age grouped by decades, for clients who were active at the end of 2019 and at the end of 2005. Percentages are calculated in column categories.

**End of 2019:**	**0-9**	**10-19**	**20-29**	**30-39**	**40-49**	**50+**	**Missing**	**Overall**	**Count**
Partial Foot	17.7	7.1	6.9	2.8	0.4	2.0	0.0	2.2	62
Transtibial	35.3	40.0	34.7	51.8	70.4	75.7	0.0	63.5	1,791
Knee Disarticulation	5.9	2.9	3.5	3.2	1.2	0.9	0.0	1.3	36
Transfemoral	5.9	22.9	33.0	31.6	22.9	17.5	0.0	19.4	547
Transradial	17.7	8.6	11.6	5.3	3.1	2.1	0.0	3.2	89
Transhumeral	11.8	4.3	5.8	2.4	1.2	0.6	0.0	1.3	36
Other	5.9	14.3	4.6	2.8	0.8	1.2	0.0	1.7	49
Missing	0.0	0.0	0.0	0.0	0.0	0.0	100.0	7.5	210
Count	*17*	*70*	*173*	*247*	*489*	*1,615*	*209*	..	*2,820*

**End of 2005:**	**0-9**	**10-19**	**20-29**	**30-39**	**40-49**	**50+**	**Missing**	**Overall**	**Count**
Partial Foot	17.2	8.1	1.6	1.4	2.2	2.4	0.0	2.1	108
Transtibial	58.6	48.3	60.6	69.3	72.2	71.7	0.0	68.6	3,546
Knee Disarticulation	0.0	2.0	1.0	0.8	0.8	0.9	0.0	0.9	46
Transfemoral	10.3	18.1	28.6	24.2	19.6	19.1	0.0	22.4	1,157
Transradial	6.9	11.4	5.3	3.0	3.1	3.3	0.0	3.6	188
Transhumeral	6.9	6.7	1.3	0.7	0.6	0.9	0.0	1.0	52
Other	0.0	5.4	1.5	0.5	1.4	1.5	0.0	1.2	60
Missing	0.0	0.0	0.0	0.0	0.1	0.2	100.0	0.3	13
Count	*29*	*149*	*678*	*2,015*	*1,623*	*665*	*11*	..	*5,170*
